# Supplementary material for: Pathophysiological Significance of Neutrophilic Transfer RNA-Derived Small RNAs in Asymptomatic Moyamoya Disease
Source: Cells. 2021 May 1;10(5):1086. doi: 10.3390/cells10051086 (PMC8147334; doi:10.3390/cells10051086)

2D Structure	Local AU	Position

```
 37                                 51
                       8mer-1a
 5' - g g T G G A - G A C C A G G G a - 3'  utr
          | : | |     | | | | | | |
 3' - u g A U C U G G U G G U C C C u - 5'  tRF
      16 15 14 13       7 6 5 4 3 2
        3' Pairing           Seed
```

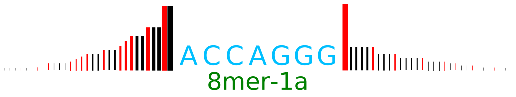

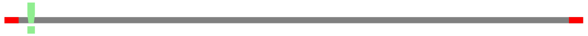

Supplement: Supplementary file 1 [file cells-10-01086-s001.zip › cells-1149161 3rd supplementary materials/Data Supplements/Data Supplement V.zip!/Data supplement 6-mRNAs targets of tsRNAs/tsRNA-Glu-CTC-003/Diagrams/tRF-Glu-CTC-003_vs_MICT00000051155(CATG00000001221.1).pdf]
